# Highest power magnification with narrow-band imaging is useful for improving diagnostic performance for endoscopic delineation of early gastric cancers

**DOI:** 10.1186/s12876-015-0385-0

**Published:** 2015-11-02

**Authors:** Kunihisa Uchita, Kenshi Yao, Noriya Uedo, Toshio Shimokawa, Takehiro Iwasaki, Koji Kojima, Ai Kawada, Mizu Nakayama, Michiyo Okazaki, Shinichi Iwamura

**Affiliations:** Department of Gastroenterology, Kochi Red Cross Hospital Japan, 2-13-51 Sinhonmachi, Kochi-city, Kochi 780-8562 Japan; Department of Endoscopy, Fukuoka University Chikushi Hospital Japan, 1-1-1 Zokumyoin, Chikushino-city, Fukuoka 818-8502 Japan; Department of Gastrointestinal Oncology, Osaka Medical Center for Cancer and Cardiovascular Diseases Japan, 3-3, Nakamichi 1-chome, Higashinari-ku, 537-8511 Osaka Japan; Graduate School of Medicine and Engineering, University of Yamanashi, 4-3-11 Takeda, Kofu City, 400-8511 Yamanashi Japan

**Keywords:** Chromoendoscopy, Demarcation line, Early gastric cancer, Magnifying endoscopy, Narrow-band imaging

## Abstract

**Background and study aims:**

Magnifying endoscopy with narrow-band imaging (ME-NBI) is more reliable than chromoendoscopy (CE) for delineating the horizontal extent of early gastric cancers prior to endoscopic submucosal dissection (ESD). However, the added benefits of ME-NBI over CE in terms of the difference in magnification level have yet to be elucidated. The aim of this study was to investigate the improvement in diagnostic accuracy for tumor delineation obtained with different magnification levels of ME-NBI following CE.

**Patients and methods:**

This was a retrospective study, performed at a single tertiary referral center. A series of 158 consecutive patients with 161 early gastric cancers resected *en bloc* using ESD was included in the study. The margins of each lesion were examined in their entirety using CE, followed by low power optical magnifying endoscopy with narrow-band imaging (LM-NBI), and finally the highest power optical magnifying endoscopy with narrow-band imaging (HM-NBI). The primary endpoint was the added benefit, as measured using the successful delineation rate, for the delineation of gastric cancer margins using CE + LM-NBI *vs* CE, and for CE + LM-NBI + HM-NBI *vs* CE + LM-NBI.

**Results:**

The successful delineation rates (95 % CI) using CE, CE + LM-NBI and CE + LM-NBI + HM-NBI were 72.7 % (68.5-79.9 %), 88.9 % (84.2-93.8 %), and 98.1 % (95.8-100 %). The diagnostic accuracy improved significantly for CE + LM-NBI compared with CE (*P* < 0.001), and for HM-NBI compared with LM-NBI (*P* < 0.001).

**Conclusions:**

HM-NBI is useful for improving diagnostic performance for endoscopic delineation of early gastric cancers, following CE and LM-NBI.

## Background

On order to be curative, endoscopic submucosal dissection (ESD) of early gastric cancers requires accurate determination of the horizontal extent of invasion so that the lesion can be completely removed in one piece [1]. In 2002, Yao et al. reported that magnifying endoscopy (ME) is useful for delineating the margins of gastric cancers [2]. The use of narrow-band imaging (NBI), developed in 2006, in combination with ME allows observation of the microvascular and microsurface patterns in high contrast [3]. A number of studies have since reported superior diagnostic ability for magnifying endoscopy with narrow-band imaging (ME-NBI) over conventional endoscopy in delineating the lateral extent of early gastric neoplasias [3–8]. The authors already use ME-NBI in the clinical setting for detection of the margins of gastric cancers, and we reported that using the maximal magnifying ration of the magnifying endoscope improves the diagnostic accuracy for delineation of the margins of differentiated early gastric cancers that could not be delineated using chromoendoscopy (CE) [4]. However, other studies have not reported the actual magnifying ratio used when performing ME [5–8].

We hypothesized that utilization of the maximal optical magnifying ratio during magnifying endoscopy provides the best spatial resolution, and increases the diagnostic ability to differentiate between cancerous and non-cancerous mucosa. However, there have been no published reports of the effect of different magnification levels on the ability to delineate margins of early gastric cancers when ME is added to CE using indigo carmine, the latter widely used clinically up to the present. The aims of this study were to determine the additive effect for the delineation of the margins of early gastric cancers, following CE, of low power optical magnifying endoscopy with NBI (LM-NBI) *vs* highest power optical magnifying endoscopy with NBI (HM-NBI).

## Patients and methods

### Patients

All investigations were performed after subjects received a thorough explanation, and provided their written informed consent. This study was a retrospective study and approved by the Ethic Committee of the Kochi Red Cross Hospital.

### Inclusion and exclusion criteria

#### Inclusion criteria

Consecutive patients with differentiated early gastric cancers, diagnosed endoscopically and assessed as suitable for ESD, and resected using ESD between July 2008 and June 2012.

#### Exclusion criteria

Lesions that could not be adequately assessed histopathologically, lesions that were not resected in one piece, and lesions with a histopathological diagnosis of undifferentiated carcinoma.

## Methods

### Examination using LM-NBI and HM-NBI

When performing ME of the upper gastrointestinal tract, once a lesion is detected using non-magnifying white light imaging (WLI), pressing the zoom lever on the endoscope control section enables examination using optical magnification. Pushing the lever down fully provides the highest power optical magnification. The focal distance at the maximal magnifying ratio is 2 mm. We defined the magnification level with a focal distance of 4 mm as low power optical magnification (LM).

**Fig. 1:**
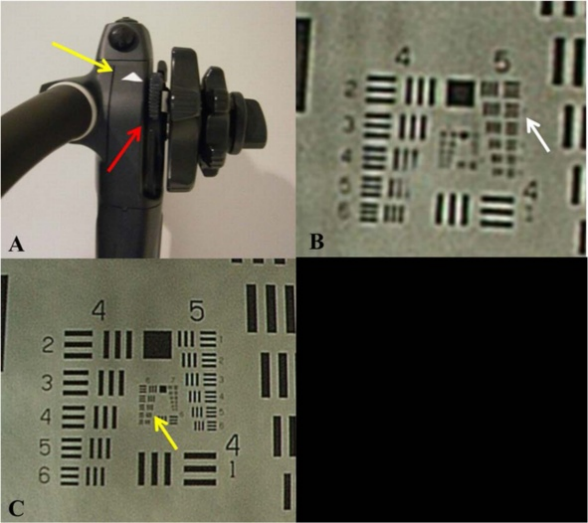
**a**. On the handle part of the scope, white marking (yellow arrow) was attached. When we press down the zoom lever (red arrow) to the point of the marking, we can consistently fix the magnification rate as low power. When we press down the zoom lever to the bottom, we can consistently fix the magnification rate as the highest power. **b**. At the low power of ME-NBI, the Line Pitch of 5–2 (white arrow) can be dissected by LM-NBI. Namely, the resolution power was measured as 13.9 μm. **c**. At the resolution power of HM-NBI, the Line Pitch of 6–4 (yellow arrow) can be dissected by HM-NBI. Namely, the resolution power was measured as 5.6 μm

To obtain this fixed LM, we made the following pre-endoscopy preparations. We attached a disposable distal attachment (D-201-11304, Olympus Medical Systems Corp., Tokyo, Japan) to the tip of the upper gastrointestinal endoscope (GIF-H260Z, Olympus Medical Systems Corp), fixing the focal distance at 4 mm. Adjusting the focus, we fixed a piece of white tape to indicate the position of the upper edge of the zoom lever, so that during an endoscopy we could depress the zoom lever to provide a consistent level of magnification (Fig. 1a). The resolutions at the low power magnification (LM) and highest power magnification (HM) were 13.9 μm and 5.6 μm, respectively (Fig. 1b, c).

### Endoscopic procedures

All endoscopies were performed by the authors, specialist endoscopists experienced with HM-NBI, using the GIF-H260Z upper gastrointestinal endoscope with the EVIS Lucera Spectrum system (Olympus Medical Systems Corp). A soft black hood (MAJ1990, Olympus Medical Systems Corp) was attached to the scope tip to obtain HM-NBI images in focus. Each patient underwent sequential CE, LM-NBI and HM-NBI examinations during the same procedure as preoperative diagnostic examinations 1 to 2 weeks prior to ESD.

### Chromoendoscopy (CE)

Following thorough lavage, the lesion was sprayed with 0.1 % indigo carmine, and the lesion margin was examined in its entirety using non-magnifying WLI.

### Low power optical magnifying endoscopy with narrow-band imaging (LM-NBI)

Following CE and thorough rinsing of indigo carmine dye, we depressed the zoom lever as far as the previously applied white tape, and examined the lesion margin in its entirety using LM-NBI.

### Highest power optical magnifying endoscopy with narrow-band imaging (HM-NBI)

Following LM-NBI, we depressed the zoom lever fully and examined the lesion margin in its entirety at the highest power magnification.

When the demarcation line (DL) could not be delineated in its entirety even using HM-NBI, we took a biopsy from the lesion surrounds in an area showing definite findings of non-cancerous mucosa, confirming that the biopsy specimen contains no cancerous tissue, thereby delineating the lesion margin over its entire circumference.

On the day of the ESD procedure, or the preceding day, using HM-NBI we again identified the lesion margin, and made markings 3–5 mm outside the DL, and then, made an incision 3–5 mm outside these markings.

Resected specimens were fixed in 10 % formalin solution. Following fixation, the specimen was sliced with 2 mm spacing, then stained for histological examination. With reference to the pathohistological findings, we identified the markings, and reconstructed the lateral extent of the cancer on the endoscopic image. The histologically determined cancer margins were used as the gold standard to evaluate the diagnostic accuracy for CM, CE + LM-NBI, and CE + LM-NBI + HM-NBI according to our previous study [4].

### Diagnostic criteria for cancer specific margins

We defined the DL as determined using CE as the area showing the two findings of “presence of a border between the lesion and non-lesion mucosa” and “disappearance of the gastric areas in the surrounding area at the border line”. We used the VS (vessel plus surface) classification proposed by Yao et al. for the definition of the DL as determined using ME-NBI. This defines the cancer margin as having the two findings of “a clear demarcation line where the surrounding regular microvascular pattern and/or regular microsurface pattern disappears” and “the presence of an irregular microvascular pattern and/or irregular microsurface pattern within the demarcation line” [4, 9–12].

**Fig. 2:**
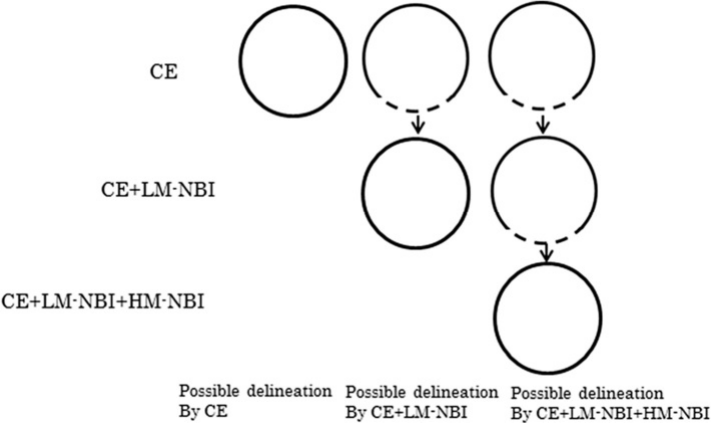
Possible lesion

**Fig. 3:**
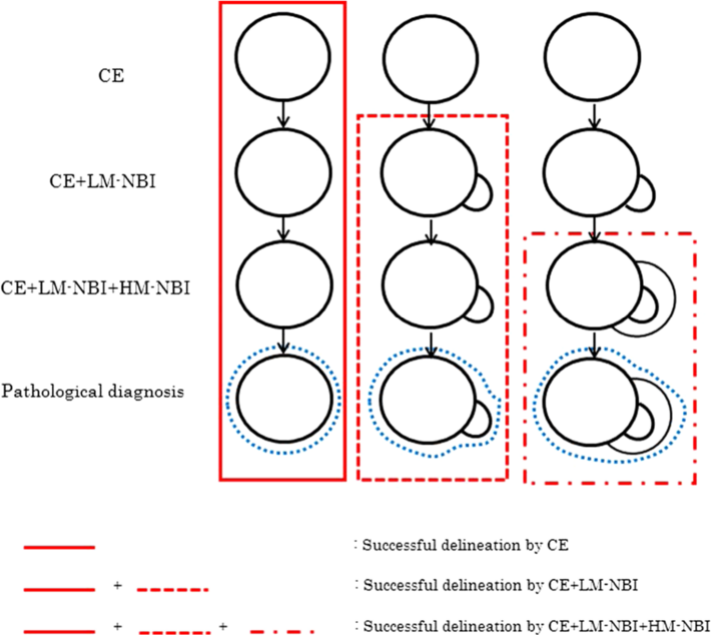
Successful lesion

For each examination method, we defined the result as “possible delineation”, “successful delineation” or “unsuccessful delineation” as follows (Figs. 2 and 3).

Possible delineation: a DL can be delineated endoscopically over the entire circumference of the lesion.

Possible delineation using CE: a DL can be delineated over the entire circumference of the lesion using CE.

Possible delineation using CE + LM-NBI: a DL can be delineated over the entire circumference of the lesion for the first time using LM-NBI.

Possible delineation using CE + LM-NBI + HM-NBI: a DL can be delineated over the entire circumference of the lesion for the first time using HM-NBI.

Successful delineation and unsuccessful delineation: defined as follows for each examination method as we reported previously [4].

Successful delineation using CE: Possible delineation using CE and, the same DL can also be delineated using LM-NBI and HM-NBI, and histological examination confirms the presence of cancer within the markings.

Successful delineation using CE + LM-NBI: Possible delineation using CE + LM-NBI, and also using HM-NBI, and histological examination confirms the presence of cancer within the markings.

Successful delineation using CE + LM-NBI + HM-NBI: Possible delineation using CE + LM-NBI + HM-NBI, and histological examination confirms the presence of cancer within the markings.

Unsuccessful delineation: a DL cannot be delineated, entirely or in part, using CE, CE + LM-NBI or CE + LM-NBI + HM-NBI, or histopathological examination of the resected specimen reveals cancer outside the preoperative markings.

The primary endpoint of this study was the added benefit, as measured using the successful delineation rate, for the delineation of gastric cancer margins using CE + LM-NBI *vs* CE, and for CE + LM-NBI + HM-NBI *vs* CE + LM-NBI. The secondary endpoint was the difference in the added benefit of each examination method according to the macroscopic type of early gastric cancer.

### Statistics

We derived the successful delineation rate with 95 % confidence intervals (CI) for early gastric cancers using each examination method, CE, CE + LM-NBI, and CE + LM-NBI + HM-NBI. We used McNemar’s test with Bonferroni’s multiple comparison correction to calculate *p* values. We also performed the same calculations for the different macroscopic types. Statistical analyses were conducted using R3.0.1. *P*-values of <0.05 indicated statistically significant.

## Results

We resected 164 lesions using ESD in 161 patients between July 2008 and June 2012. All lesions were removed in one piece, with 161 lesions in the analysis group after exclusion of 2 cases of undifferentiated carcinoma and 1 lesion that could not be assessed histologically following resection. Their clinical characteristics were as follows: average age 71 years; 116 males and 45 females; mean lesion diameter 19.2 mm (±14.4 mm, range 5–120 mm); macroscopic type using the Paris classification,　type 0-I 4 lesions (2.5 %), type 0-IIa 64 lesions (39.8 %), type 0-IIb 38 lesions (23.6 %), and type 0-IIc 55 lesions (34.2 %). The location of the lesion was the upper part of the stomach in 46 cases (28.6 %), middle part in 41 (25.5 %), and lower part in 74 (46.0 %).

**Fig. 4:**
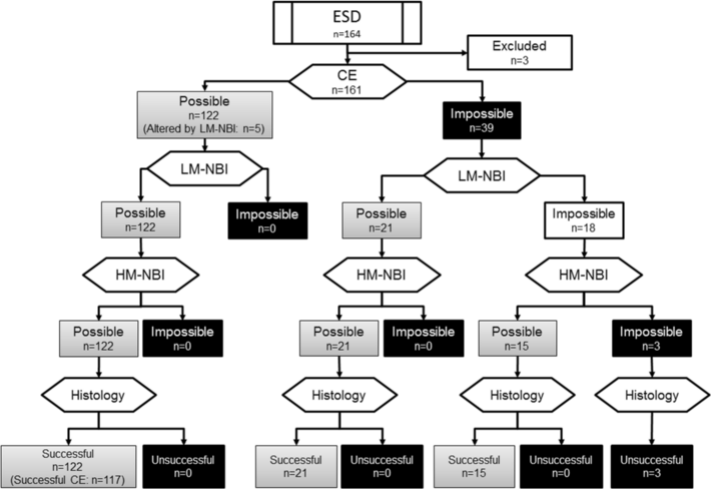
Flow diagram for the study

**Fig. 5:**
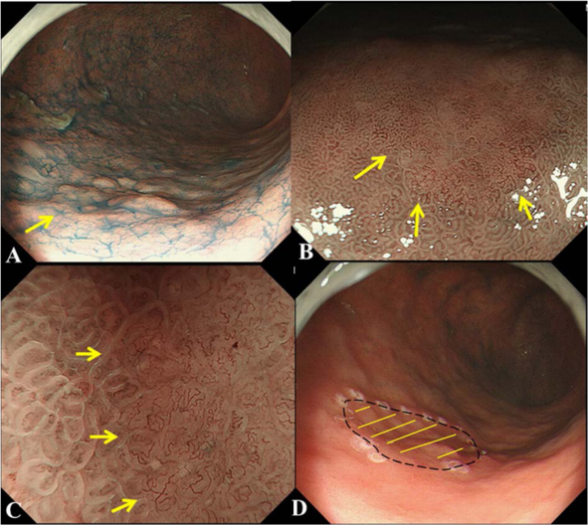
**a**. Chromoendoscopy (CE) findings of a gastric cancer of which margins of lateral extent cannot be clearly delineated until by Low-power magnifying endoscopy combined NBI (LM-NBI). A demarcation line cannot be seen by CE. **b**. LM-NBI findings of the area indicated by the arrow in **a**. A clear demarcation line can be seen. **c**. The highest-power magnifying endoscopy combined NBI findings of the area indicated by the arrow in **a**. A clear demarcation line can be seen. **d**. The extent of the carcinoma (yellow lines) was reconstructed according to the histopathological findings. The peroperative marking were clearly identified all the way around the carcinoma, as shown by black dotted line. Therefore, this lesion had been successfully delineation by CE + LM-NBI

**Fig. 6:**
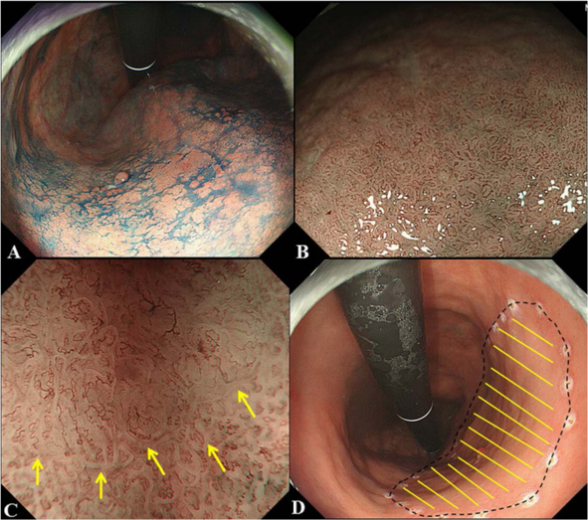
**a**. Chromoendoscopy (CE) findings of a gastric cancer of which margins of lateral extent cannot be clearly delineated until by the highest-power magnifying endoscopy combined NBI (HM-NBI). A demarcation line cannot be seen by CE. **b**. Low-power magnifying endoscopy combined NBI (LM-NBI) findings. A demarcation line cannot be seen by LM-NBI. **c**. HM-NBI findings. A clear demarcation line can be seen by HM-NBI. **d**. The extent of the carcinoma (yellow lines) was reconstructed according to the histopathological findings. The peroperative marking were clearly identified all the way around the carcinoma, as shown by black dotted line. Therefore, this lesion had been successfully delineation by CE + LM-NBI + HM-NBI

A flow diagram for this study is shown in Fig. 4. To summarize the results, the lesion DL could be identified in its entirety in 122 out of 161 lesions (75.8 %) using CE. The DL was subsequently altered in 5 lesions using LM-NBI. Accordingly, successful delineation was achieved using CE in 117 (122 – 5) lesions (72.7 %, 95 % CI 68.5-79.9 %) (Table 1). Of the 39 lesions in which the DL could not be identified in its entirety using CE, the DL could be delineated in its entirety using LM-NBI in 21 lesions, with no alterations made subsequently using HM-NBI (Fig. 5). Successful delineation was achieved using CE + LM-NBI in 143 lesions (88.9 %, 95 % CI 84.2-93.8 %), comprising the 117 cases of successful delineation using CE, as well as the 5 lesions whose DL was altered using LM-NBI, and the 21 lesions newly delineable using LM-NBI (117 + 5 + 21). The successful delineation rate improved significantly with the addition of LM-NBI (*p* < 0.001) (Table 1). HM-NBI examination of the 18 lesions in which the DL could not be identified in its entirety using CE + LM-NBI enabled identification of the DL over its entire circumference in 15 lesions (Fig. 6). Accordingly, successful delineation was achieved using CE + LM-NBI + HM-NBI in 158 lesions (98.1 %, 95 % CI 95.8 %-100 %), a further improvement in the successful delineation rate with CE + LM-NBI + HM-NBI (*p* < 0.001 *vs* CE, *p* < 0.001 *vs* CE + LM-NBI). The DL of the remaining 3 lesions could not be delineated over their entire circumferences, and they were assessed as unsuccessful delineation (Table 1). Histological examination of the resected specimens showed that all lesions remained within the preoperative markings. In other words, there were no cases of unsuccessful delineation in terms of the histological findings of the resected specimens.

**Table 1:** The number of gastric cancer with successful delineation and successful rate by chromoendoscopy and low-power magnifying endoscopy combined NBI and the highest-power magnifying endoscopy combined NBI in all lesions

	Successful delineation	Successful rate	95 % C.I
	No	%	
CE	117/161	72.7	68.5-79.9
CE + LM-NBI	143/161	88.9	84.2-93.8^a^
CE + LM-NBI + HM-NBI	158/161	98.1	95.8-100^a,b^

^a^<0.05 for *vs*. CE; ^b^< 0.05 for *vs*. CE + LM-NBI

The results of analysis according to macroscopic type are shown in Table 2. The successful delineation rate using CE for protruding lesions (n = 68) was 86.8 % (95 % CI 78.8-94.8 %), with no significant improvement in diagnostic ability seen for CE + LM-NBI (*p* = 0.06), but a significant added benefit for CE + LM-NBI + HM-NBI *vs* CE (*p* = 0.02). The successful delineation rate using CE for flat lesions (0-IIb or 0-IIb + X, n = 38) was only 34.2 % (95 % CI 19.1-49.2 %). This improved significantly to 63.1 % (95 % CI 45.0-76.0 %) for CE + LM-NBI (*p* < 0.001), and improved further to 92.1 % (95 % CI 83.5-100 %) for CE + LM-NBI + HM-NBI (*p* < 0.001 *vs* CE, *p* < 0.004 *vs* CE + LM-NBI). The successful delineation rate using CE for depressed lesions (n = 55) was 81.8 % (95 % CI 71.6-92.0 %), improving significantly to 98.2 % (95 % CI 94.6-100 %) for CE + LM-NBI (*p* = 0.02). The successful delineation rate for CE + LM-NBI + HM-NBI was 100 % (95 % CI 100 %), showing a significant additive effect vs CE (*p* = 0.01). Accordingly, for all macroscopic types HM-NBI significantly improves the diagnostic ability of endoscopic examinations, most notably in flat lesions where morphological changes are often subtle. This study also showed that for flat lesions HM-NBI further improves the diagnostic ability over LM-NBI.

**Table 2:** The successful rate in each macroscopic type by chromoendoscopy and low-power magnifying endoscopy combined NBI and the highest-power magnifying endoscopy combined NBI

Macroscopic type	CE	CE + LM-NBI	CE + LM-NBI + HM-NBI
Elevated (0-I, 0-IIa)	86.8 % [78.8-94.8]	97.1 % [93.1-100]	100 % [100]^a^
Flat (0-IIb, 0-IIb + X)	34.2 % [19.1-49.2]	63.1 % [45.0-76.0]^a^	92.1 % [83.5-100]^a,b^
Depressed (0-IIc)	81.8 % [71.6-92.0]	98.2 % [94.6-100]^a^	100 % [100]^a^

^a^<0.05 for *vs*. CE; ^b^< 0.05 for *vs*. CE + LM-NBI

## Discussion

ESD has become the standard endoscopic treatment for early gastric cancer, and endoscopic treatment is indicated for all histologically differentiated intramucosal cancers, regardless of size, as long as there is no ulceration [13–16]. Accordingly, accurate delineation of the horizontal extent of gastric cancers, and minimizing the extent of the resection is important in minimizing invasiveness for the patient, as well as making things easier for the endoscopist. Magnifying endoscopic examination at the highest power magnification provides the best spatial resolution, which enables endoscopists to visualize microvascular pattern which cannot be sufficiently achieved by LM-NBI. Therefore, we can assume that it also allows optimum accuracy for delineation of gastric cancer margins. However, a search of the literature failed to yield any studies of changes in diagnostic ability for different magnification levels of ME-NBI. In this study, the diagnostic rate for delineation of gastric cancer margins was 72.7 % for CE, 88.9 % for CE + LM-NBI, and 98.1 % for CE + LM-NBI + HM-NBI. In other words, margin delineation using HM-NBI following CE yielded the highest diagnostic ability. This study, with strict setting of the endoscopic magnifying ratio, for the first time demonstrated an added benefit for the magnification level in accurate delineation of the margins of early gastric cancers in order to achieve successful endoscopic resection of early gastric cancer even for 0-IIb type.

CE enhances the contrast between areas with different topography on the mucosal surface, but some early gastric cancers are completely flat (type 0-IIb), making margin delineation particularly difficult with conventional non-magnifying endoscopy. Mishima et al. reported that from a series of early gastric cancers resected using ESD, the margins were unclear using CE in 48 % of type 0-IIb lesions [17]. Furthermore, Nagahama et al. reported a successful delineation rate of 81.8 % using CE, compared to our rate of 72.7 %. A reason for this difference may be that the Nagahama series only included 8 % of type 0-IIb lesions, less than our figure of 23.6 % for the proportion of type 0-IIb lesions [4]. Our results also yielded high successful delineation rates for CE of 86.8 % for protruding lesions and 81.8 % for depressed lesions, but extremely low at 34.2 % for flat lesions. ME-NBI for delineation of the margins of type 0-IIb lesions allows us to assess not only changes in the surface structure, but also changes in the microvasculature, thereby improving the diagnostic ability. Other studies of delineation of the margins of type 0-IIb lesions using ME-NBI have reported successful delineation rates of 61 % (Oyama et al.) and 75 % (Kobayashi et al.) [18, 19]. These are similar to the successful delineation rate obtained in this study using CE + LM-NBI, whereas the successful delineation rate for CE + LM-NBI + HM-NBI of 92.1 % was markedly higher than those in the Oyama and Kobayashi studies. The reason for this may be that we performed HM-NBI in all cases.

The mechanism whereby HM-NBI further improves the diagnostic ability is likely as follows. With ME-NBI, we diagnose gastric cancers through examination of the gastric mucosal microvascular pattern (MVP) and microsurface pattern (MSP). The MSP can be adequately visualized using LM-NBI, but the resolution of approximately 14 μm for LM-NBI is insufficient to discern mucosal microvessels (MVs) with a minimum diameter of 8 μm. Visualization of the MVP, a requirement for the accurate diagnosis of gastric cancers, is therefore impossible with low power magnification. The resolution of HM-NBI is 5.6 μm, however, providing sufficient information to adequately assess MVs and accurately diagnose gastric cancers [3].

The main limitation of this study was that it excluded undifferentiated gastric cancers. Nagahama et al. performed ME-NBI in all cases, reporting a lower successful delineation rate for undifferentiated than for differentiated gastric cancers [4]. Further studies of diagnostic ability that include undifferentiated gastric cancers are required. The other limitation is that for this retrospective study have there is a possibility that we could not cancel the carrying-over effect because we observed the target lesion by HM-NBI following LM-NBI. In this study, we examined the added benefit of the magnification level, and did not directly compare the diagnostic ability of LM-NBI and HM-NBI. Further elucidation of the effect of magnification level on diagnostic ability will require comparison of the results of LM-NBI and HM-NBI as independent procedures. Examinations using the highest power magnification level are technically difficult, and experience is needed to produce clear images. Future studies of reproducibility of results, with multiple endoscopists experienced with highest power magnification levels at multiple institutions, should also be conducted.

In conclusion, ME-NBI is an extremely useful modality for the delineation of the margins of gastric cancers, and HM-NBI is useful for improving diagnostic performance for endoscopic delineation of early gastric cancers, following CE and LM-NBI.
